# Meteo-Hydrological Sensors within the Lake Maggiore Catchment: System Establishment, Functioning and Data Validation

**DOI:** 10.3390/s21248300

**Published:** 2021-12-11

**Authors:** Marzia Ciampittiello, Dario Manca, Claudia Dresti, Stefano Grisoni, Andrea Lami, Helmi Saidi

**Affiliations:** Water Research Institute, National Research Council, 28922 Verbania, Italy; marzia.ciampittiello@irsa.cnr.it (M.C.); dario.manca@cnr.it (D.M.); claudia.dresti@irsa.cnr.it (C.D.); grisoni.stefano@gmail.com (S.G.); andrea.lami@irsa.cnr.it (A.L.)

**Keywords:** hydro-meteorological stations, sensors, web services, PITAGORA project

## Abstract

Climate change and human activities have a strong impact on lakes and their catchments, so to understand ongoing processes it is fundamental to monitor environmental variables with a spatially well-distributed and high frequency network and efficiently share data. An effective sharing and interoperability of environmental information between technician and end-user fosters an in-depth knowledge of the territory and its critical environmental issues. In this paper, we present the approaches and the results obtained during the PITAGORA project (Interoperable Technological Platform for Acquisition, Management and Organization of Environmental data, related to the lake basin). PITAGORA was aimed at developing both instruments and data management, including pre-processing and quality control of raw data to ensure that data are findable, accessible, interoperable, and reusable (FAIR principles). The main results show that the developed instrumentation is low-cost, easily implementable and reliable, and can be applied to the measurement of diverse environmental parameters such as meteorological, hydrological, physico-chemical, and geological. The flexibility of the solutions proposed make our system adaptable to different monitoring purposes, research, management, and civil protection. The real time access to environmental information can improve management of a territory and ecosystems, safety of the population, and sustainable socio-economic development.

## 1. Introduction

In the evaluation of the impacts of climate change and human activities on lakes and their catchments, accurately monitoring environmental parameters, both in spatial and temporal terms, is increasingly important for water resource management, quality assessment, and protection [1,2]. The national and international market does not present flexible and low-cost instrumentation that is necessary for a continuous and large-scale monitoring of lakes and their catchment areas, and it is unclear whose data can be adequately shared by various stakeholders such as institutional authorities, environmental protection agencies, tourist institutions, and citizens [3]. A network focused on the development and application of innovative sensor technology called NETLAKE currently exists. This network encourages the realization of homemade meteorological and limnological sensors, because they are more flexible for different research needs, more manageable, low cost, and easy to implement [4]. Additionally, the Global Lake Ecological Observatory Network, GLEON, is aimed at responding to the need for scientific understanding of lake processes, supporting the use of advanced technologies of different types of sensors [5]. The sensors used in this network are both commercially produced and built by the individual research groups, according to their different specific needs [6]. However, the specific needs of research in the environmental field are above all linked to: (i) the collection of high-frequency data that are not readily available in commercial instruments, (ii) the possibility of changing the acquisition frequency, (iii) continuously improving performance, and (iv) a data processing software and inoperability that commercial tools offer only in some cases and with high costs [7]. Furthermore, the sharing and the interoperability of environmental information, individual data, or boundary information constitute the basis for an in-depth knowledge of a territory, with its peculiarities and criticalities and therefore of its potential for sustainable, eco-compatible, deep, and lasting development in time [8]. Good data stewardship is essential to maximize data access and reuse, and to ensure reproducibility in plant sciences as indicated by FAIR (Findability, Accessibility, Interoperability, and Reusability) principles [9]. Consequently, the first point to be addressed for testing the implementation of a measurement network is the choice of an area of greatest meteorological, environmental, and socio-economic interest [10]. The presence of meteorological–hydrological stations, establishing them and making them an active and functional part of a process of improvement of knowledge of the environment, grows awareness and social sensitivity with respect to its vulnerability and potential and the need for sustainable economic development [11]. The second point is developing innovative technological solutions that are able to manage the acquisition of signals from environmental sensors, conduct local storage and basic processing of raw data, and transmit wirelessly in real time or near real time the information and data [12,13]. It is important to consider that real time processing requires continual input, constant processing, and steady output of data. Examples of real time processing are data streaming, radar systems, and webcams. In near real time processing the frequency of transmission is important, but processing time in minutes is acceptable in lieu of seconds. In general, near real time processing are sensors data and IT system monitoring. In our case study, pluviometers and webcams are considered in real time and all the other sensors in near real time.

For this reason, to deepen environmental and meteorological knowledge and their evolution under the effects of climate change and human activities, during 2014–2015 a regional project was developed, funded under the POR FESR 2007/2013 of the Piedmont Region with the assistance of community FESR resources, the Italian State, and the Piedmont Region. This project’s name (an Italian derived acronym) was PITAGORA—Interoperable Tehcnological Platform for Acquisition, Management and Organization of Environmental data, related to lake basins (In Italian: “Piattaforma Interoperabile Tecnologica per l’Acquisizione, la Gestione e l’Organizzazione dei dati Ambientali”). The aim of this project was to develop a participatory governance system for lake resources, based on innovative technology. 

PITAGORA was a project based on integrated lake basin management (ILBM); this concept convenes the political and scientific communities to merge their vision and coordinate their actions to contribute to improved ecosystem governance [14]. The partnership of the project consisted of four small-medium enterprises (SME) and three research institutions. One of the activities developed in the framework of this project by our Institute (CNR IRSA/ISE, Verbania), was the realization of an ICT system based on the Internet of Things. This system was conceptualized for the protection and management of the aquatic ecosystems of Lakes Maggiore, Orta, and Mergozzo (Northwest of Italy, Piedmont Region) and their rivers basins, with the definition of an integrated system for the measurement, acquisition, transmission, and use of environmental data. This ITC system made data and information available to different end users: directors, tourists, citizens, and students through the practical application of the European INSPIRE legislation, with the dissemination of OGC standards to ensure the interoperability of the data collected. The purpose of this paper is to present the approaches, the results, and the instrument developed during the PITAGORA project and to present the subsequent advancement work on data acquisition, data transmission, web services, and elaboration of raw data. The system created by assembling the sensors and the hardware made in-house, and managed according to the recent European legislation on interoperability and transmission, is considered innovative as it responds to different research needs by applying different protocols and solutions and it also functions in remote areas. The system created with PITAGORA is therefore unique in its kind as other similar networks have not faced the problem of raw data and their processing. The developed system is adopted in other fields like hydrogeology, through the construction of geophones, and limnology, through the construction of fixed and portable buoys, all realized in perfect accordance with the FAIR principles.

## 2. System Description

**Figure 1:**
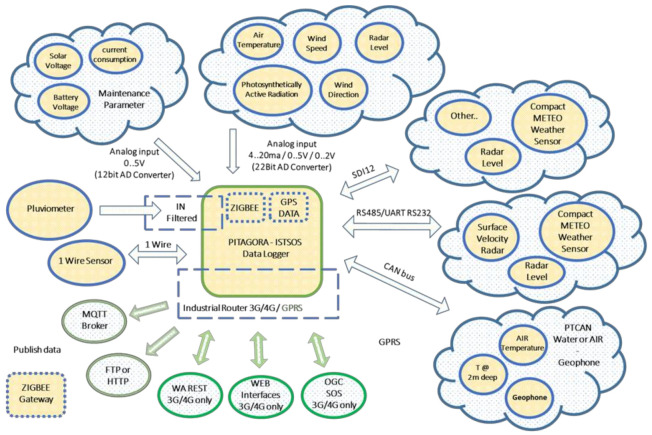
Scheme of PITAGORA system.

An open source hardware approach was chosen for building a digital device, which can be used by other users for similar applications; each can develop specific software applications and add new boards in appropriate expansion slots. Figure 1 shows the general scheme of the PITAGORA system. Part of the system comes from commercial hardware chosen from the most recent and best suited to our technological needs, such as sensors, router, GPS, and GPRS. Other components are instead the result of settlement and new creations made in house, such as (i) the input IN FILTER of the rain gauge (to control electromagnetic interference), (ii) the high precision water or air temperature with CAN BUS, (iii) the motherboard (green square in the Figure 1), and (iv) the interconnections between the various parts of the system. In particular, thank to our system, it is possible to choose a suitable internal communication technology to use, analog or digital depending on the sensors, and what kind external communication to adopt, depending on the presence of specific infrastructure for data transmission in the station area and if it located in remote site or not (for example, wireless communication). Furthermore, having chosen the best sensor for each environmental parameter to be measured, it was necessary to define different communication modes among them and the motherboard, for example, by inserting 12 and 22 bit converters. Finally, it is also possible to use different data management protocols, analog and digital such as SD12, UART, CAN bus, and 1 Wire. 

A more detailed description of the adopted system is given in the supplementary material (Figures S1–S4) where we present a description of motherboard (the green square in the Figure 1), SDI-12—serial digital interface, UART—universal asynchronous receiver-transmitter (RS485/UART RS232), 1 Wire, CAN-bus—controller area network, transmission, analog input voltage, GPS, GPRS, ZIGBEE, and power supply.

Below are some examples of hydro-meteorological stations realized during the PITAGORA project, placed in different locations, with their different equipment.

**Figure 2:**
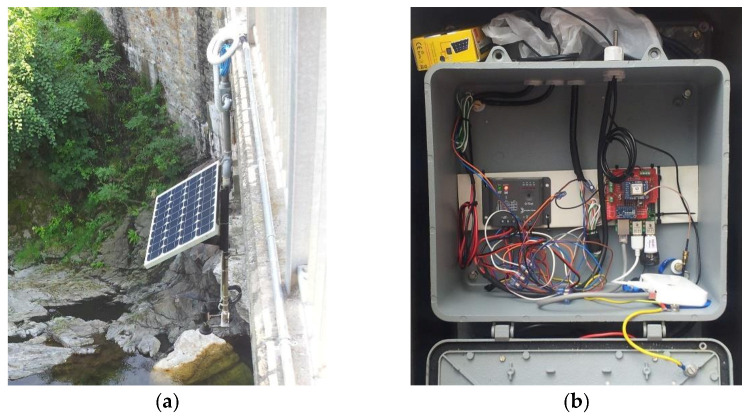
Hydrological station on River Cannobino: (**a**) hydrological sensor and solar panel; (**b**) detail of the contents of the shell: modem and various electronic elements.

In Figure 2a,b, is pictured the hydrological station placed on the Cannobino River, one of the main tributaries of Lake Maggiore. It is one of the few rivers into the Lake Maggiore catchment that maintains a natural discharge. The new frequency of collected data and the possibility of having data in near real time offers the possibility of closely monitoring flood events and/or the effect of prolonged periods of drought.

**Figure 3:**
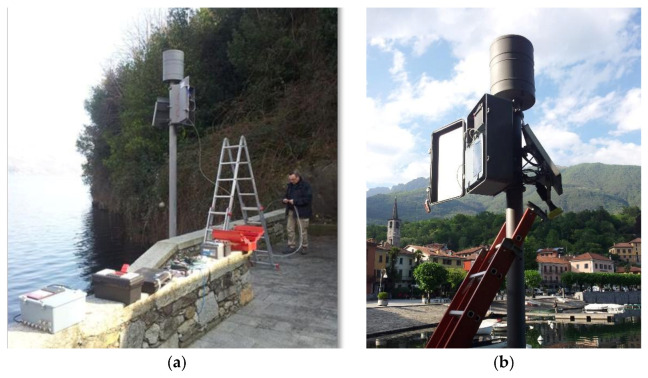
Hydro-meteorological station of Lake Mergozzo: (**a**) upgrade of Mergozzo hydro-meteorological station with new sensors and IOT electronic board; (**b**) main elements of hydrometeorological station of Lake Mergozzo: pluviometer, robust shell that contains electronic elements, solar panel, and hydrometer.

In Figure 3a,b is pictured the hydro-meteorological station placed on Lake Mergozzo that measures rainfall, air temperature, and lake level. The data collected with the new system allow the collection of hydro-meteorological information also useful for tourists.

**Figure 4:**
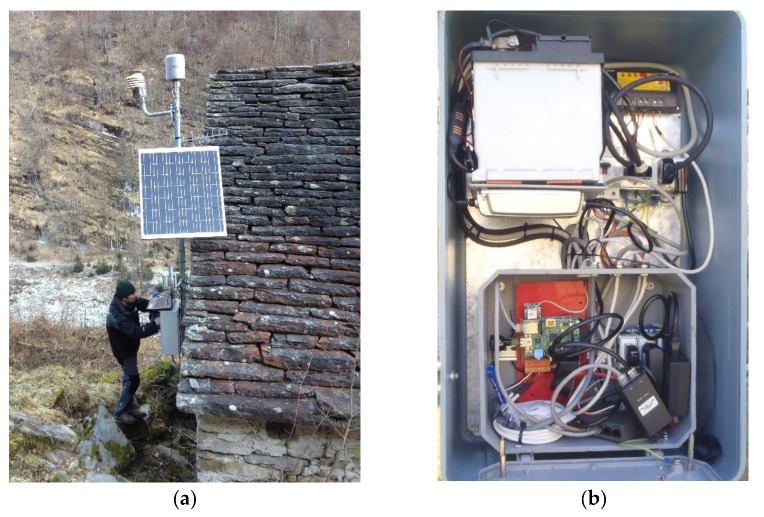
Meteorological station of Craveggia: (**a**) pluviometer, solar panel, and robust shell; (**b**) detailed contents of the shell: battery, modem, various electronic elements.

In Figure 4a,b, is pictured the meteorological station of Craveggia, which was placed in a location that is particularly difficult to reach, with little irradiation and very low temperatures in winter.

### 2.1. Web Services

The increasingly widespread awareness of the importance of having reliable, precise, easy-to-maintain, low-cost, and easily interfaced and interoperable measuring instruments, whether they are new generation or have been on the market for some time, has opened the way to checks on the correct instrumental functionality. This development led to new needs for the management of the instrumentation, for the exchange of data, and for the definition of common standards for the use of information and services, available on the web and usable by everyone [15] (http://www.opengeospatial.org (accessed on 6 December 2021)). The idea of developing new technological structures for the acquisition and dissemination of information and environmental knowledge was therefore born, aimed not only at the detection of data in the field, but also at their transmission in real time and near real time, and at the visualization, through web services, both of the measured data (submitted to validation) [16] and of the information relating to the previous trends of some critical parameters (such as, for example, the fluctuation in the level of a river, the amount of rain in a given period of time, the variation of indicator parameters of the chemical-physical or ecological quality of a water body [17]). One criticism highlighted concerns about the discovery of available datasets, and another one concerns the interoperability of data with other sources, together with the quality of the data and their control [18]. Hence, the need to define common characteristics both in the description of the datasets and in the way of presenting them. To reach this objective, the European community launched the INSPIRE directive that defined a legal framework to describe metadata and environmental data, also with the aim of supporting the drafting of policies that can have a direct or indirect impact on the environment. [19,20]. Other globally recognized organizations such as the Open Geospatial Consortium (OGC) have defined common languages to display such data. The OGC is an international organization that aims to develop standards for content, services, and the exchange of geographic data. Among the standards proposed by OGC we can note Sensor Web Enablement (SWE) [21], which includes access to sensors and their observations, alert systems, and data exchange. The functionalities that OGC has set as a goal for the Sensor Web are: (i) access to the sensors, their observations, and the processing of the observations themselves; (ii) determination of the capabilities of the sensor and the quality of its measurements; (iii) access to the sensor parameters to allow the software to geolocate and automatically process the observations; (iv) retrieval of observations in real time and near real time and by time series, encoded in standard formats, and (v) criteria for error handling by sensors or sensor-based services [22,23].

The architecture used for the service interfaces, which are used for geographic information and the definition of relationships with the Open Systems Environment model, is the ISO19119 standard: “Geographic information—Services” (https://www.iso.org/standard/59221.html (accessed on 6 December 2021). It defines the generic architecture of services for a geographic information management system according to ISO international standards. It also prescribes how to create a platform-neutral service specification, how to derive conformant platform-specific service specifications, and provides guidelines for the selection and specification of geographic services from both platform-neutral and platform specific perspectives [24]. The creation of METADATA was according to the ISO 19115 standard: “Geographic information—Metadata” (https://www.iso.org/standard/26020.html (accessed on 6 December 2021), which provides information about the identification, extent, quality, spatial and temporal aspects, content, spatial reference, portrayal, distribution, and other properties of digital geographic data and services; for example, it is possible to catalog all types of resources, clearinghouse activities, and the full description of datasets and services, geographic services, geographic datasets, dataset series, and individual geographic features and feature properties.

Another usable web service that has been standardized is presented by the descriptive, structural, administrative, statistical, and spatial reference information of the collected data. All this information is collected in a catalog called “METADATA repository”. Metadata standards are specifications that intend to define a common understanding of the meaning or semantics of data [25] in such a way as to ensure their interpretation and the correct use by their owners and other users [26] data and to make their retrieval easier [27]. However, metadata takes on less importance when data collected remain within a narrow scope and in the case of direct data exchange. That is the case of the PITAGORA project, for which we decided to not consider metadata. Currently we are trying to insert the PITAGORA metadata catalog on a platform such as D4Science Infrastructure (https://www.d4science.org/ (accessed on 6 December 2021). D4Science is an open science oriented e-Infrastructure [28]. The advantage of this open-source system is that it is able to process big data and offers free to use processes as a service from multiple domains. For the future development of our project, D4Science will provide us with a web-based set of facilities including data and computational facilities to accomplish a set of tasks, and it will foster the collaboration between project partners working on the same topic while managing data.

The Observation & Measurements (O&M) standard, one of the core standards in the OGC Sensor Web Enablement suite, providing the response model for Sensor Observation Service (SOS), has been implemented in our system and allows the XML encoding of observations and measurements acquired by sensors both in real time and near real time and stored in the archive [29] (https://www.ogc.org/docs/is (accessed on 6 December 2021). 

For the OGC standard the observations are based on the following five classes or entities: (i) Procedure, the sensor (electronic or analog); (ii) Observed Property (quantities to be measured or observed); (iii) Result (the observation or value of the measurement); (iv) Sampling time (data and time when the observation or measurement was carried out), and (v) Feature of interest (place or point where the observation or measurement is made, identified by its coordinates).

The Sensor Model Language (Sensor ML) was implemented, which allows the description of the sensor networks and the processes associated with the measurements. The purposes of this architecture are to provide the description of sensors and sensor systems; to provide the description of the process used by the sensor to make an observation; to support the geolocation of sensors; and to provide performance-related characteristics (e.g., performance, threshold).

The Sensor Observation Service (SOS) was implemented, as it represents the standard web service for querying, filtering, and displaying information from sensors. This service acts as an intermediary between any client and a recorded data repository or a near real time data transmission channel. This standard is used in the case of interoperable collected data. SOS is based on the OGC Observation & Measurements (O&M) standard to encode the observations collected by the sensors.

Another interesting web service is the 52° North Sensor Observation Service that is an interoperable interface for inserting, querying, and extract sensor data and metadata. It manages live in-situ sensor and historical datasets (https://52north.org/software/software-projects/sos/ (accessed on 6 December 2021) We had implemented at the beginning of the PITAGORA Project this service but, very early, we realized that it was very complex to extract data and to work with raw data. Indeed it is a very useful service for exchanging data or putting together data between and from different sources and different institutions and if you want to build applications to query data that are not managed directly, but, if you need to manage the data directly from the machine it is very complicated and laborious. Therefore, we decided to use only CSV files to transform, transmit, and store our data, which are more appropriate to save, retrieve, and describe data from hydro-meteorological sensors. In our project, to get data from a hydro-meteorological station we use csv files that contain the raw data.

The RESTFul interface is a webservice through which a data server is asked to return information. This interface offers better access from any device and grants the access to the collected time-series graphs and observation data using HTTP operation calls into the URLs. 

### 2.2. Data Acquisition

The basic characteristics of a meteorological measurement station are reliability of operation, measurement accuracy, construction simplicity, robustness, and ease of calibration and maintenance [30]. Most of these characteristics are possessed by the sensors at the time of purchase, others (constructive simplicity, robustness, and ease of calibration and maintenance) must be achieved by integrating the sensors with the other components of the weather station (e.g., CPU, communications.). The frequency of acquisition of the measurement by the various sensors depends on the use of instrumental sensitivity, the data itself, whether for research, civil protection, or ecological analysis purposes [31].

In our study and for our research interests, the frequency of acquisition selected and the accuracy of the each sensor are:

- For air temperature: ten acquisitions are made per minute, an average is calculated, and this value is recorded. The daily maximum and minimum values are calculated. In addition the time (hour and minute) at which these values (maximum and minimum) were recorded is indicated. The measurement range is –30 °C, +60 °C, the accuracy ±0.2 °C. For sensors that couple temperature and humidity, the measurement range for temperature is –40 °C, +80 °C and for humidity is 0–100%, the accuracy ±3% if relative humidity is between 10% and 90%, ±5% if relative humidity is <10% and >90%.
- For rainfall: at the beginning one datum per minute was measured and the average, minimum, and maximum values every 5 min were recorded; during the development of hydrometeorological stations we changed this mode to have more information on data features and more flexibility on subsequent data analysis and so now it evaluates a single tipping event and takes the timestamp of it. We know that for each tipping event it records a rainfall value, for our instruments, equal to 0.2 mm. The measurement range is 0–300 mm/h, the accuracy ±3%.
- For wind speed and direction: we have wind sensors with both analog and digital output. In the first case (analog output) we have created a protocol that, by continuously reading the values, (approximately 60/70 acquisitions per minute), returns, every minute, the average of the measurements, both regarding the gusts and the direction.
- Digital sensors, on the other hand, provide an already converted numerical response. For wind speed the measurement range is 0–75 m/s, the accuracy ±2% @12 m/s, the response time 0.25 s with resolution equal to 0.01 m/s. For wind direction the measurement range is 0–359°, the accuracy ±2° @12 m/s, the response time 0.25 s with resolution equal to 1°.
- For solar radiation: we used a thermopile solarimeter or thermopile pyranometer that detect incident global solar radiation. It is a first class standard instrument; one datum per minute is registered, therefore 1440 data per day. The raw data are then processed directly. The measurement range is 0–1500 W/m^2^, the accuracy ± 5%, spectral window 305–2800 nm.
- For lake and river level: one datum per minute is measured and the average values every 5 min were recorded. The daily maximum and minimum values were calculated. The measurement range is 0–15 m, the accuracy 2 mm.

With regard to the humidity, the sensor is inserted in the sensor coupled with the temperature sensor, so we have chosen to use thermohygrometers; regarding the pressure sensor we have not yet positioned it in a meteorological station equipped with this sensor.

## 3. Validation and Elaboration Tools

The environmental features measured by the sensors may be subject to sudden deviations from their average values or their expected values, just as any malfunction of the sensor or of the data acquisition-transmission-storage infrastructure is always possible. It is therefore of primary importance to define control protocols and verify the validity of the collected/measured data, both starting from the acquisition infrastructure placed in the field, and subsequently, in more detail, within the storage platform [32]. A control of the measured values on several levels gives greater certainty of the validity of data, their actual variability, and their scientific value within statistical analyses or specific elaborations, such as in the evaluation of the daily and/or seasonal trends of meteorological quantities such as rain or air temperature. The first check is inside the acquisition instrumentation, using simple verification values such as instrumental thresholds, historical or absolute maximum or minimum values, and simple comparisons between trends of quantities, so that through a first screening it is possible to identify big errors and/or malfunctions. A second level of validation of the meteorological-hydrological data, more specific and in-depth, is then carried out within the platform using both the previous historical data available through calculation methods and ad hoc models for the definitive validation of the measured data. These methods and models take into account the pre-screening already carried out and are therefore prepared for any errors and the reconstruction of the data, if necessary, as well as to validate the data collected also with the comparison of nearby stations and therefore through space-time processing.

According to the provisions of the WMO, the procedures for quality control resulting from the commission of experts were standardized and published as guidelines [33,34,35]. Within these guidelines it is recommended to use different quality control procedures at different stages of data acquisition and storage, implementing analysis methods at all levels, from in situ, directly on weather stations, to those within the “Data Processing Center”.

In addition, spatial validation is also required that considers not only the specific measurement station but also the presence, within a territory, of previous information from other stations. The coding of errors associated with individual data must be part of the information surrounding each datum and quality control procedure.

To date, the structuring of good quality, robust sensors with low costs and a low cost of realization of a remote management of a data acquisition-transmission-archiving system that also contains calibration, control, and validation procedures has not yet been fully realized [36]. The main purpose of carrying out a quality control of the data is the establishment of a robust database, fed by correct values of environmental quantities, which can be used for statistical and predictive analyses and models for forecasting trends over time and space of the measured meteo-hydrological parameters.

The errors that normally have to be evaluated in the quality control procedures can be summarized as follows:

- Random errors: these are errors symmetrically distributed around zero that do not depend on the measured value. Sometimes these errors are overestimated, sometimes underestimated with respect to a real expected value. The average of the measured values generally eliminates this type of error.
- Systematic errors: these are distributed asymmetrically with respect to zero; on average, these errors tend to generate an error in the measured values, either higher or lower than the actual value and over time, if not corrected, causing a “drift” in the measurement.
- Gross errors: these are caused by sensor malfunctions and therefore show obvious errors or gaps in the measurement; they are easily identifiable both at the level of verification of the raw data and subsequently in the processing phases.
- Microscale errors: these are due to small perturbations on small measurement, temporal, and spatial scales. If this type of error occurs during routine observations, the result appears quite inhomogeneous and extraneous compared to observations and measurements made at the same time in nearby stations or in successive time intervals.

As an example, the consistency between the values and/or the trend of two parameters connected to each other is evaluated, for example, it is verified that the maximum temperature is greater than the minimum temperature; that if the wind speed is zero so is the direction; that if the measured rainfall is zero so is its cumulative value; that the humidity decreases as the temperature increases; that solar radiation is zero at night, that if it rains the relative humidity cannot be less than 80%.

The quality checks subsequent to that carried out directly on the measuring device are performed within the PITAGORA platform, and in particular concern random, systematic, and microscale errors. These types of errors can only be identified through the processing of data on an hourly, daily, and monthly scale, as it is only through a comparison over time of the collected data that such errors can be identified as correctly as possible. With regard to microscale errors, it is also important to make a comparison with data collected from nearby stations, which is only possible for a platform where a certain number of both historical and territorial data are present.

The control rules that can be used within the platform can be summarized as follows:

- Checks on the correct spatial-temporal attribution of data;
- Checks on the consistency of the data recorded by each individual instrument;
- Checks on the consistency of the time series;
- Cross-checks on different instruments in the same station;
- Cross-checks on the same instruments in the different stations.
- The procedures that can be used for the validation at the last level of the collected data are:
- B3: QA/QC tool for sensor data (http://gleon.org/research/projects/b3-a-qaqc-tool (accessed on 6 December 2021);
- RClimtool (https://cgspace.cgiar.org/bitstream/handle/10568/63483/RClimTool_UserManual.pdf?sequence=3&isAllowed=y (accessed on 6 December 2021).

The first quality control that was carried out concerns gross errors, that is, a verification of acceptance or otherwise of plausible values; a second first-level check was carried out in the case of meteorological data for internal consistency between the values of various parameters, as indicated in Table 1, in order to use clear internal consistencies to identify major anomalies.

## 4. Discussion and Work in Progress

The principle of the project was to purchase the sensors and electronic components separately, in order to assemble a multi-parameter system that can be adapted to specific needs, which can be implemented and modified over time and at very low costs. Sensors were selected to be rugged enough to withstand prolonged use in the field. A totally scalable solution was therefore implemented, capable of supporting increasing volumes of data according to the paradigms of big and fast data scenarios [37,38].

The model conceived and created is highly dynamic, thanks to data collection, trans-mission, and processing tools so as to have:

- Greater flexibility of the instrumentation with the possibility of interacting with the sensor in the field to program specific acquisitions/calibrations;
- Possibility to implement the instrumentation, adding one or more sensors to the one or more already present, easily and with little expense;
- Reduction of management costs;
- Standardization of the data format.

This technological solution was necessary both for the climate change impact and for the numerous anthropic activities on the lakes and their catchment, and because the national and international market, while offering many different types of sensors, has not yet adapted to the new European regulations, and above all, does not present such flexible and low-cost instrumentation as that necessary for a continuous and large-scale monitoring of lakes and their catchment [39]. Additionally, the traditional methods for storing meteorological data and the web services for these data do not meet the needs of high performance and the amount of in real-time big data and processing [40].

Regarding aspects more related to the data acquisition and transmission system, as well as information disclosure services, the current position is:

- Automate the transmission, possibly releasing it from telephone operators;
- Develop web services that can be used by various stakeholders, including interactive ones and ones associated with data processing software;
- Remotely manage alarms and out of range values.

The sensors can acquire data from a fixed or mobile electronic station through special serial interfaces that allow the future insertion of new sensors as needed. The station transmits data in “unsolicited” mode to the data proxy in near real time mode or at regular intervals, as needed [16]. 

The sensor network operates according to a model that guarantees robustness with respect to connection availability. To do this, the paradigm known as store and forward [41] is used, according to which the measurements are always stored locally (on the station) and sent to a server immediately or as soon as the connection is active. The electronic station is equipped with a GPS module with geolocation and date and time synchronization functions and a robust power supply system with batteries and multiple power sources to ensure autonomous operation [42]; the status of the power system is also transmitted to allow preventive maintenance of the station.

Therefore, intelligent stations were developed as they are capable of verifying the validity of the measured data, sending data when possible, identifying hydro-weather warnings and malfunctions, and consequently sending alert signals. 

**Figure 5:**
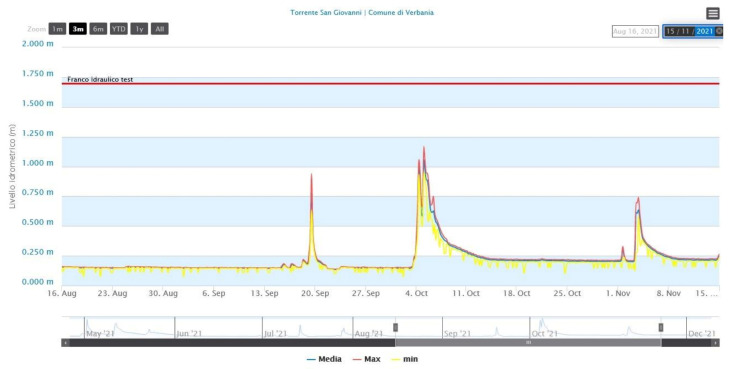
Web representation of the near real time trend of river level during the last 3 months. Blue line represents mean values, red lines maximum levels, and yellow line minimum levels.

Between the end of 2020 and the beginning of 2021, a collaboration between our Institute and the Retemet Company (https://www.retemet.com/en/ (accessed on 6 December 2021) began, which led to the activation of meteo-hydrological stations in the Liguria Region, in order to test and refine our acquisition technologies and data transmissions in the meteorological-hydrological field and to obtain improvement feedback. From this collaboration a friendly output of the hydro-meteorological data was born, an example of which is shown in Figure 5 (https://www.navimeteoharbour.com/app/blowing/idrometro_core_history.html?id=sangiovanni (accessed on 6 December 2021).

The implementation of the architecture, services, and system created through the PITAGORA project continued afterwards, verifying the flexibility and adaptability of this infrastructure to other fields in addition to the specific hydro-meteorological one.

**Figure 6:**
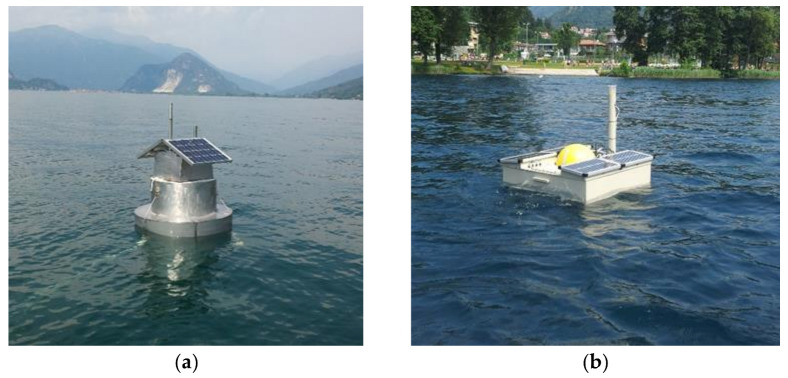
Limnological buoys: (**a**) fixed buoy on Lake Maggiore; (**b**) mobile (portable) buoy on Lake Orta.

In particular, both fixed and mobile limnological buoys were built and deployed in the study lakes during the PITAGORA project (Figure 6) and after, with the SIMILE project (Integrated monitoring system for knowledge, protection and valorization of the subalpine lakes and their ecosystems), an Interreg Italian-Swiss project funded by the European Regional Development Fund (ID 523544). (https://www.progetti.interreg-italiasvizzera.eu/it/b/78/sistemainformativoperilmonitoraggiointegratodeilaghiinsubriciedeiloroe (accessed on 6 December 2021)). 

**Figure 7:**
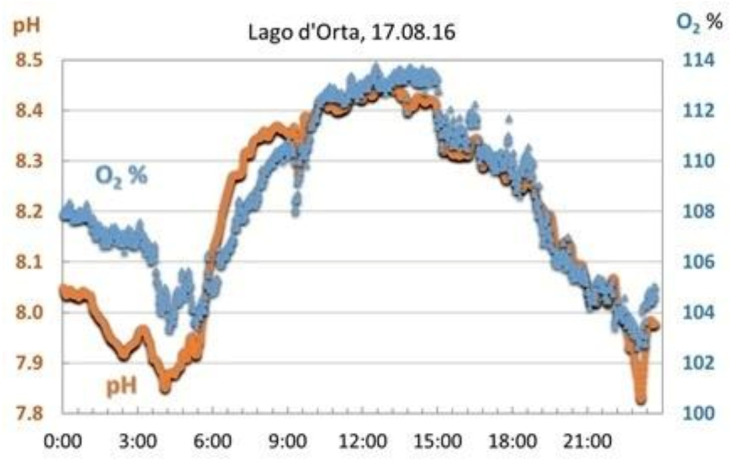
Example of how the high-frequency collection of limnological parameters can increase the degree of knowledge of the responses of a lake ecosystem. Graph shows hourly fluctuation of pH and oxygen.

The buoys collect high frequency data of basic limnological parameters such as water temperature, pH, electrical conductivity, and dissolved oxygen. These data allowed an assessment of rapid changes in limnological variables and in-lake processes (Figure 7). High frequency (HF), near real time data integrate traditional monitoring (e.g., discrete lake sampling), providing information on processes occurring on short time scales. These data are useful to detect rapid changes in physical, chemical, and biological parameters and may provide new insights into processes that drive change.

Furthermore, as part of a collaboration between our Institute and CNR IRPI (National Research Council - Research Institute for geo-hydrological Protection) aimed at carrying out geo-hydromorphological and environmental research and monitoring activities in the field of triggering factors, dynamics, and evolution of instability processes along slopes and the hydrographic network, experimental systems were created for the multiparametric acquisition of hydro-geo-morphological indicators of initiation and dynamics of debris flows, further strengthening the existing monitoring instrumental network. We developed an experimental system consisting of various sensor and electronic components: a geophone (2016 to date).

**Figure 8:**
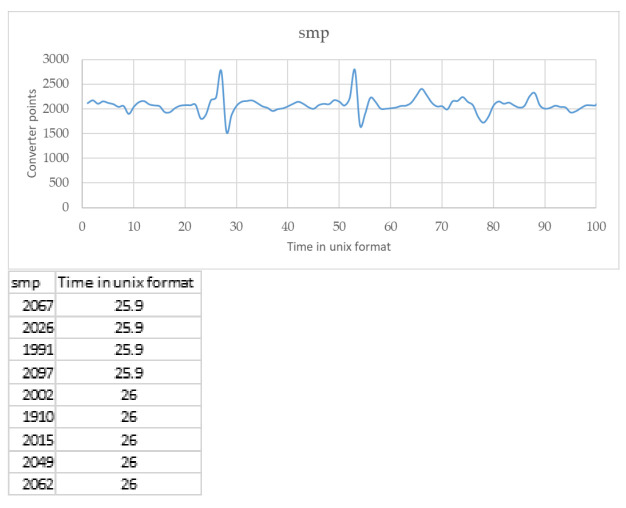
Example of graph and data outcome from geophones. The count starts at the Unix Epoch on 1 January 1970. Smp are the converter points, the number that the instrument gives, its analog signal.

The geophone is a sensor capable of detecting ground movements or even seismic waves. The sensitive element is similar to a microphone, capable of detecting very low frequencies of even a few Hertz, and transforming this signal into an electrical impulse, which is then represented with graphs and tables (Figure 8). Our geophone works with a frequency equal to 2 Hz. 

The management system of our automated weather stations will be based on istSOS software [43]. In addition, we will use an open science oriented e-Infrastructure like D4Science to optimize access to data sources and to meet the principles of transmission, management, and accessibility of hydro-meteorological data.

## 5. Final Remarks

Since 2014, the intense laboratory and field activities for the implementation and optimization of hydro-meteorological stations, targeted at being low cost, easily implementable, flexible, reliable, intelligent, standardized, and technologically advanced has led to the realization of several tools for environmental monitoring and research.

We tested that the instrumentation developed can be applied to the measurement of quite diverse environmental parameters such as meteorological, hydrological, physico-chemical, and geological. The possibility of using this instrumentation for different purposes (monitoring, research, civil protection) offers a better and a more in-depth knowledge of climatic and ecological dynamics, as well as geo-hydrological evolution. The greater the “environmental” knowledge, the better the chances of developing activities that bring economic, cultural, and scientific advantages for a whole territory in which the possibility of accessing real time and near real time meteo-hydrological and physical-chemical data can become crucial for the management of any emergencies. Only through technologically advanced systems that are easy to use and maintain it is possible to guarantee a correct management of the territory and ecosystems, for the safety of the population and more sustainable socio-economic development.

## Figures and Tables

**Table 1:** Validation criteria for the control unit that were adopted, including those parameters for which there are no experimental data yet (pressure).

Parameters	Invalidation Criterion	Flag
Precipitation	I < 0 (mm/min) I > 40 (mm/min)	Erroneous
Temperature	T< −40 °C T > + 60 °C	Erroneous
	∆T(per minute) > 3 °C	Suspect
	∆T(per hour) < 0.1 °C	Suspect
Wind velocity	V < 0 m/s V > 50 m/s as an average value	Erroneous
	V > 50 m/s as maximum gust	Erroneous
	∆V (per minute) > 20 m/s	Suspect
	∆V (per hour) < 0.5 m/s	Suspect
	V = 0 m/s and direction is not zero	Suspect
Wind direction	Wind direction < 0 Wind direction > 360 degrees	Erroneous
	∆Wind direction (per hour) < 10 degrees	Suspect
	Wind direction = 0 and V is not zero	Suspect
Pressure	P < 940 hPa P > 1060 hPa	Erroneous
	∆P (per minute) > 0.5 hPa	Suspect
	∆P (per hour) < 0.1 hPa	Suspect
Humidity	H < 0% H > 100%	Erroneous
	∆H (per minute)> 10%	Suspect
	∆H (per hour) < 1%	Suspect
	H = 0% with non-zero precipitation	Suspect
Solar radiation	Solar radiation <0 Solar radiation > 900 W/m^2^	Erroneous
	Solar radiation > 0 during the night	Erroneous
	∆ Solar radiation (per minute) > 900 W/m^2^	Suspect

## Data Availability

The authors confirm that the data and the material supporting the findings of this study are available and could be requested from the authors upon reasonable request to the corresponding author.

## Supplementary Materials

The following are available online at https://www.mdpi.com/article/10.3390/s21248300/s1, Figure S1: PITAGORA motherboard, equipped with Plug-IN RS484/422 board and analogue inputs board, Figure S2: Photograph of the board and diagram of 1 Wire for pluviometer, Figure S3: Diagram of RS485 standard, Figure S4: Scheme of power supply elements [16,44,45,46,47,48,49,50,51,52,53,54,55,56,57,58,59,60,61,62,63,64].
