# Supplementary material for: Meteo-Hydrological Sensors within the Lake Maggiore Catchment: System Establishment, Functioning and Data Validation
Source: Sensors (Basel). 2021 Dec 11;21(24):8300. doi: 10.3390/s21248300 (PMC8705426; doi:10.3390/s21248300)
Supplement: Supplementary file 1 [file sensors-21-08300-s001.zip › sensors-1460872-supplementary.pdf]

Meteo-hydrological sensors within the Lake Maggiore catchment: system establishment, functioning and data validation

Marzia Ciampittiello[1], Dario Manca[1], Claudia Dresti[1], Stefano Grisoni[1], Andrea Lami[1] and Helmi Saidi[1,*]

1 Water Research Institute, National Research Council, Italy; marzia.ciampittiello@irsa.cnr.it; dario.manca@cnr.it, claudia.dresti@irsa.cnr.it; grisoni.stefano@gmail.com; andrea.lami@irsa.cnr.it; helmi.saidi@irsa.cnr.it
* Correspondence: helmi.saidi@irsa.cnr.it

Supplementary materials

System description

The electronic control unit that deals with the acquisition of signals from environmental sensors, local storage, and basic processing of raw data and sending of information wirelessly, which has been processed, has the following architecture:

- Motherboard – It is the core of the system and it is specified by the green square in Figure 1 in the main text. In summary, its functioning, and its constituent elements are: low voltage power supply connection with relative fuses; battery connection with relative fuses; battery management card; 5VDC power supply and 3V3 for CPU Beagle Black or RaspberryPI CPU board; sensor data acquisition cards – serial; ADC input cards with high resolution and precision 0-10V and 4-20mA; generic output card with relay or open collector transistor; wireless communication card; generic cards for future use; photovoltaic solar panel management card; electronics for serial line multiplex; basic electronics. In Figure S1 is a photograph of one realized motherboard:
- Ten connectable sensors with possible expansion;
- Lead acid or LiFePO4 battery;
- Networking is provided by a built-in USB Ethernet adapter or any external communication devices (such a generic modem, GPRS , radio ,etc) with a serial UART232 cable;
- Possible 230Vac main power supply.

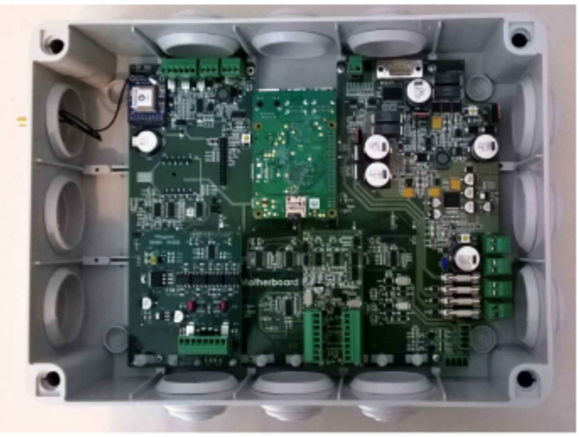

Figure S1. PITAGORA motherboard, equipped with Plug-IN RS484/422 board and analog inputs board.

This kind of architecture can be used also in geographically isolated areas, thanks to programmable data-acquisition in the sleep mode with the possibility to control performance and efficiency during long periods and to optimize future systems [16].

The control unit is able to acquire signal from analog and digital sensors where the output is documented by the manufacturer. The protocol or standard managed are:

- SDI-12—Serial Digital Interface at 1200 Baud:

This is an asynchronous serial communications protocol for intelligent sensors that monitor environment data. These instruments are typically low power (12 volts), are used at remote locations, and usually communicate with a data logger or other data acquisition device [44]. The protocol follows a master-slave

configuration whereby a data logger (SDI-12 recorder) requests data from the intelligent sensors (SDI-12 sensors), each identified with a unique address [45].

Electrically the protocol is a 3wire digital connection: data, ground, and 12V. The data signal, using 5V logic levels, is similar to RS-232 with the same asynchronous byte encoding. The inline data are human readable as the data are transmitted in ASCII.

All SDI-12 communications are transmitted in ASCII at 1200 baud with 7 data bits and an even parity bit. The standard also specifies a communications protocol that allows sensors to remain in a low-power sleep state until awoken by a serial break signal sent by the master. The first character of each command is a unique sensor address that specifies with which sensor the recorder wants to communicate. Other sensors on the SDI-12 bus ignore the command and return to low-power standby mode. The protocol also specifies a retry mechanism to overcome any data corruption. CRCs were introduced to the SDI-12 protocol with release of version 1.3.

- UART—Universal Asynchronous Receiver-Transmitter:

This is a block of circuitry responsible for implementing serial communication. Essentially, the UART acts as an intermediary between parallel and serial interfaces. It is a general purpose or dedicated hardware device. This device facilitates serial transmission and reception of data [46].

It converts data bit streams from parallel format to asynchronous serial format or vice versa. The UART signals are expected to be converted into physical communication standards: TTL / RS232 / RS485 / RS422 [47].

- 1 Wire:

1 Wire is the only voltage-based digital system that works with two contacts, data and ground, for half-duplex bidirectional communication (Figure S2). A 1-Wire system consists of a single 1-Wire master and one or more 1-Wire slaves. The 1-Wire concept relies both on a master that initiates digital communication and on self-timed 1-Wire slave devices that synchronize to the master's signal [48]. The timing logic of master and slave must measure and generate digital pulses of various widths. When idle, a high-impedance path between the 1-Wire bus and the operating voltage puts the 1-Wire bus in the logic-high state. Each device on the bus must be able to pull the 1-Wire bus low at the appropriate time by using an open-drain output (wired AND) [49]. If a transaction needs to be suspended for any reason, the bus must be left in the idle state so the transaction can resume.

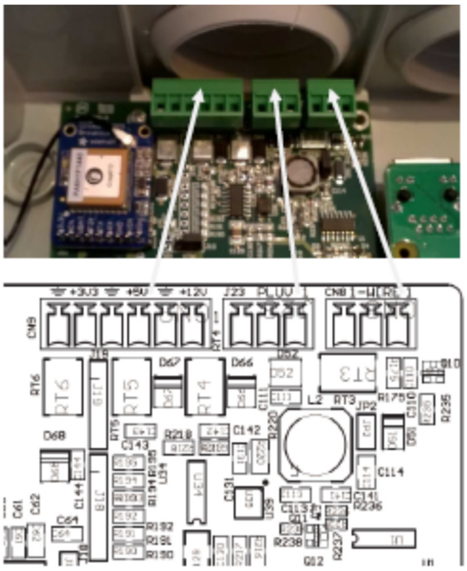

Figure S2. Photograph of the board and diagram of 1 Wire for pluviometer.

- CAN-bus—Controller Area Network:

This i a serial standard for fieldbus (mainly in the automotive environment), of the multicast type, introduced in the 1980s by Robert Bosch GmbH [50], to connect various electronic control units (ECUs). The CAN has been expressly designed to work without problems even in environments strongly disturbed by the presence of electromagnetic waves and can use a balanced potential difference line such as RS-485 (Figure S3) as the transmission medium [51]. EMC noise immunity can be further increased by using twisted pair cables.

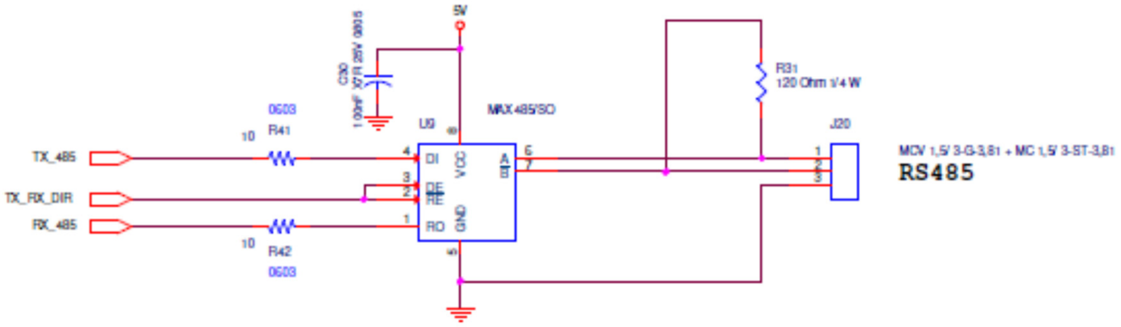

Figure S3. Diagram of RS485 standard.

- 4–20mA Current Transmission:

The 4–20mA current loop is widely used as an analog communication interface in industrial applications for transmitting the data from remote sensors. Four milliamps represents the lowest temperature value, and 20mA represents the highest measured value [52]. There are four main advantages of the current loop. First, the voltage drop in the loop does not affect the accuracy of the signal as long as the power-supply voltage is greater than the total voltage drop across the loop. Second, it uses two wires for power as well as data communication over the entire loop. Third, it is immune to noise. Finally, it is offered at a low cost and easy installation [53].

There is a current / voltage converter with relative high precision A / D conversion (16bit) via Chip MAX1301 communicating with the Beagle card via SPI channel.

The input is provided with a low noise and ESD protected MAX9632 amplifier.

- 0–10V Analog Input Voltage:

Analog sensors convert the value of the measured variable into an electric signal, so that sensors communicate their values in 0–10V voltage; an intelligent sensor contain a microprocessor that converts the measured value of the variable into a digital signal for direct communication to other intelligent devices [54].

A high-precision A / D converter (16bit) is provided via Chip MAX1301 communicating with the CPU card via SPI channel [55]. The input is provided with a low noise and ESD protected MAX9632 amplifier [56]. This amplifier can operate in a very wide supply voltage range. The exceptionally fast settling time and low distortion make this an excellent solution for precision acquisition systems. The rail-to-rail output swing maximizes the dynamic range when driving high-resolution, even with low supply voltages [57].

A serial bus consists of just two wires, one for sending data (TX) and another for receiving (RX). There are all sorts of standards for serial signalling; ones of the more popular hardware implementations of serial is transistor-transistor logic level (TTL) [58].

The control unit is able to control different communication devices through serial TX/RX TTL port communication.

The GPS circuit is always present with the task of geolocation and synchronization of the date and time; it is used to know the accuracy of the position and velocity information produced by the hydro-meteorological sensors [59].

It is expected to be battery-powered so that in the event of a shutdown, the restoration of the functions is immediate (one second).

The GlobalTop Technology Inc. PA6H GPS Module with external / internal antenna is used. It communicates in serial TX / RX TTL to the CPU card through a software module specification.

The wireless communication technologies are widely applied in the fields like industrial automation, home area network, and monitoring systems. Bluetooth, X-10, Wi-Fi, and Zigbee are the preferable wireless technologies for application in these areas. Bluetooth and Zigbee are the wireless technologies for low data collected in monitoring and control activities [60].

Zigbee is an IEEE 802.15.4-based specification for a suite of high-level communication protocols used to create personal area networks with small, low-power digital radios, such as for home automation, meteo and

limnological device data collection, where low-power low-bandwidth needs, designed for small scale projects which need wireless connection [61].

XBEE and XBEE-PRO from DIGI industries are used.

GPRS communication has great advantages, such as real-time online transmitting, rapid logon, easy monitoring, safety, and reliability. GPRS performed well above all meteorological data transmission even if interrupted suddenly or frequently. A GPRS circuit also offer the possibility of data transmission from hydro-meteorological stations, when a cellular mobile telephone communication network is available [62].

GPRS expands the functionality of GSM-based data exchange services, providing:

- PTP service (Point-to-Point): interconnection between internet networks (IP protocol) and networks based on X.25;
- Point-to-Multipoint (PTM) service: group calls and multicast calls;
- MMS Messaging (Multimedia Messaging Service).

The module chosen for communication is SIM900, utilized in areas with no internet connection [62]. It communicates in serial TX / RX / RTS / CTS TTL and there is no audio communication. This module is expected to be in a state of low absorption with a consumption of 30uA via an external signal command or AT command. As for the antenna, a connection for an external antenna is provided.

The module is supplied with continuous energy (between 3.2 and 5 V) and absorbs a maximum of 0.8 A during transmission.

In remote sites, where typically no cellular communication network is present, a radio modem has the task of communicating the data coming from the sensors. This architecture needs a base station that is installed in the presence of a network. The base station is mainly responsible for receiving the data from the sensing unit and then transferring them to the PC or the server in the network [63]. The modem is connected via RS232 serial communication and powered at + 12V or + 5V with the possibility of ON / OFF control from the CPU board. The RS232 serial communication is commonly adopted to accomplish the latter task, due to its simplicity [64]. The module family managed are driven using the Standard AT Command.

The control unit is designed to have autonomy even in isolated locations where no electricity sources exist. It is therefore envisaged that as energy input it is possible to connect:

- 12VDC power supply from mains (when available);
- Solar panel max 100W. Typical data of a 100W Polycrystalline panel;
- Another energy source capable of delivering a voltage of about 14VDC such as a little Eolic generator.

It is present the control of:

a) Input voltage and current from the solar panel;
b) Input voltage and current from 13.8VDC power supply;
c) Lead acid battery voltage and current;
d) LiFePO4 battery voltage and current.

These values will be sent by the post office as status information.

The control takes place through the direct ADC lines available from the CPU Board suitably adapted to have a maximum input of 1.8V. The conversion is at 12Bit and therefore at 4095 intervals (Figure S4).

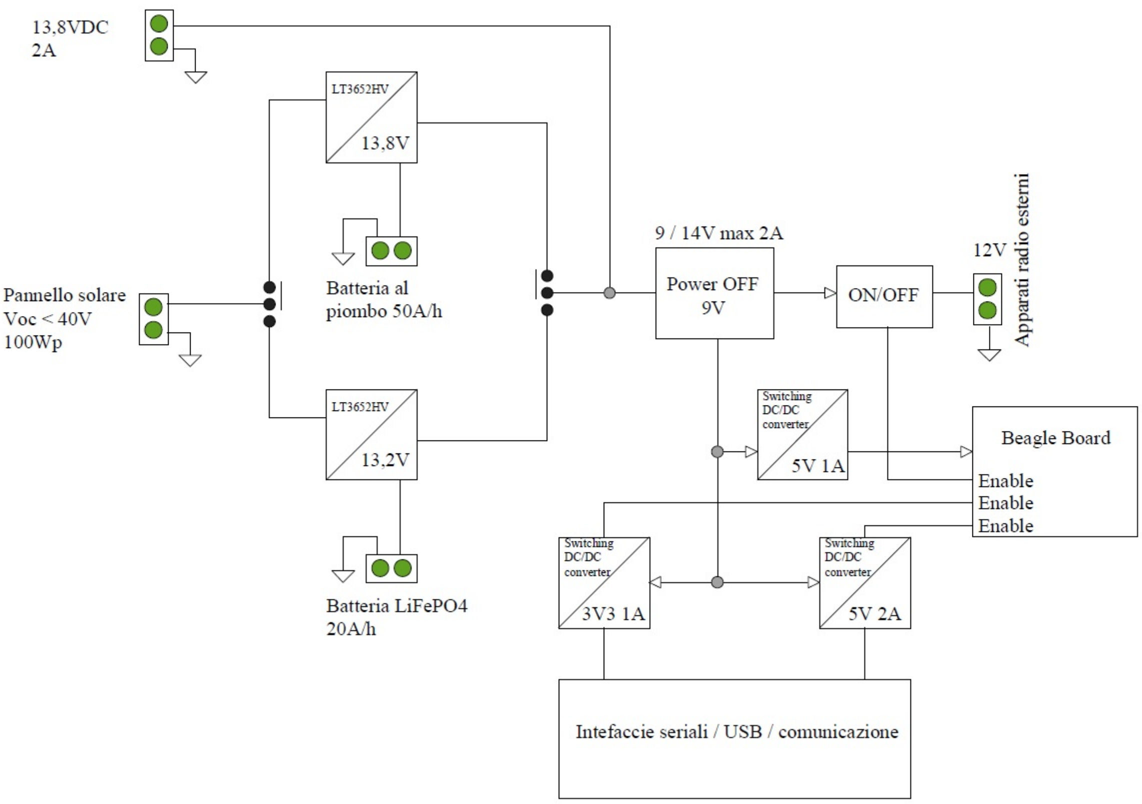

Figure S4. Scheme of power supply elements.
